# Biomechanical investigation of tasks concerning manual materials handling using response surface methodology

**DOI:** 10.1038/s41598-023-43645-2

**Published:** 2023-10-04

**Authors:** Amit M. Adhaye, Dhananjay A. Jolhe, Akshay R. Loyte, Yuvarajan Devarajan, Subash Thanappan

**Affiliations:** 1 Department of Mechanical Engineering, Visvesvaraya National Institute of Technology, 440010, Nagpur, Maharashtra India; 2 Department of Mechanical Engineering, Saveetha School of Engineering, SIMATS, Saveetha University, Chennai, Tamilnadu 602104 India; 3 Department of Civil Engineering, Ambo University, Ambo, Ethiopia

**Keywords:** Engineering, Mathematics and computing

## Abstract

In typical manual material handling, the variations in walking pattern are decided by various factors, such as load being handled, frequency of handling, walking surface, etc. Traditional gait analysis protocols commonly evaluate individual factor within specified ranges associated with particular activities or pathologies. However, existing literature underscores the concurrent impact of multiple factors on gait. This study identifies five pivotal factors—walking speed, surface slope, load carried, carrying method, and footwear—as contributors to gait alterations. To address risk factors in manual material handling activities, we propose a unique design-of-experiment-based approach for multi-task gait analysis. Unraveling the relationship between manual handling attributes and human gait holds paramount importance in formulating effective intervention strategies. We optimized the five input factors across a cohort of 15 healthy male participants by employing a face-centered central composite design experimentation. A total of 29 input factor combinations were tested, yielding a comprehensive dataset encompassing 18 kinematic gait parameters (such as cadence, step length etc., measured using inertial measurement system), the isolated impacts of factors, and the interplay of two-factor interactions with corresponding responses. The results illuminate the optimal scenarios of input factors that enhance individual gait performance—these include wearing appropriate footwear, employing a backpack for load carriage, and maintaining a moderate walking pace on a medium slope with minimal load. The study identifies walking speed and load magnitude as primary influencers of gait mechanics, followed by the chosen carrying method. In consequence, the insights gained advocate for the refinement of manual material handling tasks based on the outcomes, effectively mitigating the risk of musculoskeletal disorders by suggesting the interventions for posture correction.

## Introduction

The human body is a complex biological structure built through bones, muscles, ligaments, tendons, and blood^1^. The capability of musculoskeletal system to work/carry out certain activities is based on its structure and strength. However, poorly designed manual work often causes strain on human body, which in longer term leads to musculoskeletal disorders (MSDs). Work-related musculoskeletal disorders (WMSDs) are prevalent in manual material handling (MMH) involving improper postures, load beyond safe limit and high frequency of handling^2^. It is found that work-related musculoskeletal disorders are the second most common reason for global disability^3^. The relationship between work-related musculoskeletal disorders and the productivity of human resources has been explored by many researchers such as Manjunatha et al.^4^; Paul et al.^5^; Ray et al.^6^.

Various ergonomic assessment techniques are employed to investigate the risk of manual activities on human health and to improve overall productivity in the workplace. A few of the recent applications of such techniques can be found in Brandl et al.^7^, Enez & Nalbantoğlu^8^, Garg et al.^9^, Haekal et al.^10^, Meepradit et al.^11^, Pispero et al. ^12^, Ramadhani et al.^13^. As evident from the work of Battini et al.^14^, Joshi & Deshpande^15^, Rajendran et al.^16^, the results obtained through these ergonomic tools are mostly qualitative and insensitive to minor variations in the posture. This highlights a need for employing quantitative tools in the assessment of manual material handling tasks.

The present study deals with critical evaluation of a manual handling activity using gait analysis technique. Gait analysis helps to quantify the change in an individual's walking pattern and associated abnormalities. Whittle^17^ and Saunders et al.^18^ have defined the normal and abnormal gait which help to trace the sources of such deviations in gait. These studies further recognize the kinematics gait parameters as the important gait determinants. Pelvic tilt, pelvic obliquity, knee flexion–extension, ankle mechanism, foot mechanism and lateral displacement of body are the six gait determinants combinedly results into a much smoother trajectory for the center of gravity and a much lower energy expenditure. Kaufman & Sutherland^19^ presented the normal range of kinematic gait parameters applicable to certain age group. The gait parameters are broadly classified as spatio-temporal, kinematic and kinetic. Adhaye and Jolhe^20^ emphasized the numerous gait variables that have been taken into account by researchers for risk identification in diverse applications. Kinematic gait parameters such as step length variability, hip range of motion (ROM), knee ROM and trunk ROM increased with respect to fatigue and load applied^21^. The joint kinematics are capable of detecting the gait deformities such as (i) Trendelenburg gait (lateral trunk bending) and waddling (by assessing pelvic obliquity and hip adduction-abduction), (ii) anterior / posterior trunk bending (by assessing pelvic tilt and hip flexion–extension), (iii) excessive lumbar lordosis and hip joint ankylosis (by assessing step/stride length, pelvic tilt and hip flexion–extension), (iv) circumduction, hip hiking, steppage and vaulting (by assessing pelvic symmetry ⁒, pelvic obliquity, pelvic rotation, flexion–extension at the hip, knee and ankle), (v) abnormal hip rotation (by assessing ankle eversion-inversion)^17^. Gait pattern, characterized by multiple gait parameters, is affected by many factors, such as walking speed^22^, weight being carried^23–26^, load and fatigue^21^, footwear^27^, walking surfaces^28^, hip contact forces^29^, carrying methods^23^, viewing angle, clothing, walking surface settings and time elapsed^30^ etc. There is limited literature citing interactive effects of factors responsible for gait altercations. In addition to single factor effect, two-factor interaction effects must be considered while designing the intervention strategies for gait improvement.

In the present research, effect of five factors; footwear, load carrying method, load handled, slope of walking surface, and speed of walking on the gait pattern associated with a specific manual material handling activity is explored. Response Surface Methodology (RSM) is employed to comprehend individual factor effects and two-factor interaction effects of these five factors on the 18 gait parameters. The results obtained helped to optimize the input factors so as to minimize the risk of musculoskeletal disorders to the workers.

## Method

**Figure 1:**
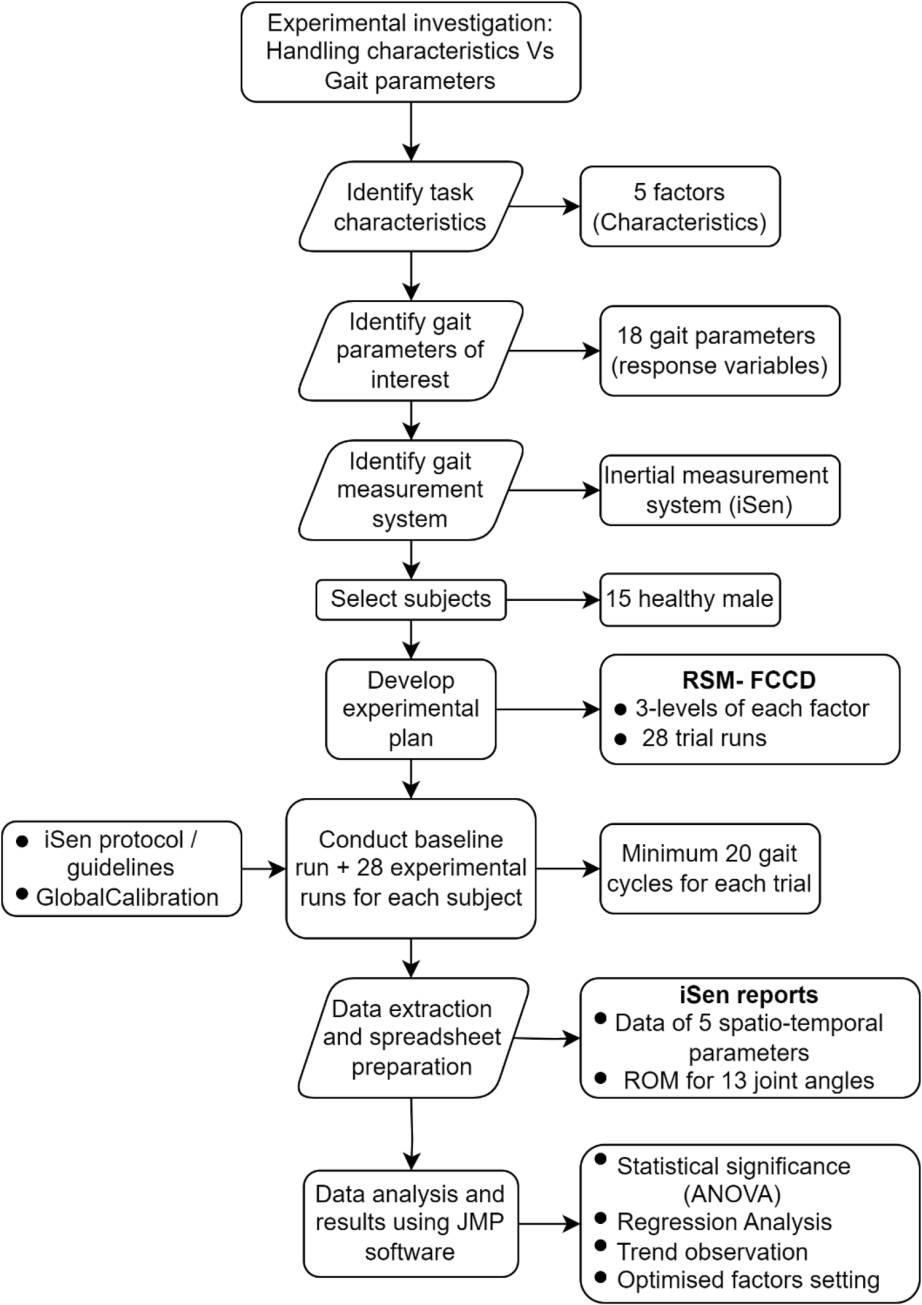
Experimentation flowchart.

The experimental methodology adopted here is depicted in Fig. 1 and the steps adopted are discussed in the following sub-sections.

### Activity under consideration

The manual material handling activity under investigation involves handling of grain bags at a food warehouse. The activity typically consists of lifting/lowering the bags and walking along levelled/sloped/irregular surface in unloaded (UL) and loaded (L) conditions. Huge number of workers are engaged in this labour-intensive activity with an average of 130 bags being handled per person per shift of 8 h. The bags are lifted and carried on either back or shoulder by the workers. Thus, a cycle of manual material handling activity consists of lifting on the back (LoB) and carrying on the back (CoB) or lifting on the shoulder (LoS) and carrying on shoulders (CoS). As an interventional strategy to reduce impact of weight of bag on the back, a specially designed backpack was introduced. This inducted a third method of carrying, i.e., carrying on backpack (CoBp).

### Subjects

The participants comprised of 15 healthy male subjects selected randomly from the workers involved in the activity. The number of subjects for experimentation were selected based on the sample size calculated using equation for continuous variable^31^. The demographic data of the subjects selected for the study is shown in Table 1. The purpose of the study was explained to the subjects in advance, and informed consents were sought. The subjects were reported to be free from injuries, illness, or musculoskeletal disorders that could affect their gait patterns.

**Table 1:** Demographic data of the workers involved in the study.

	Age (Year)	Height (m)	Weight (kg)	BMI
Range	26–52	1.54–1.80	40–90	15–32
Mean	37	1.66	61.21	22.10
SD	10	0.067	11.21	3.86

### Response surface methodology

**Figure 2:**
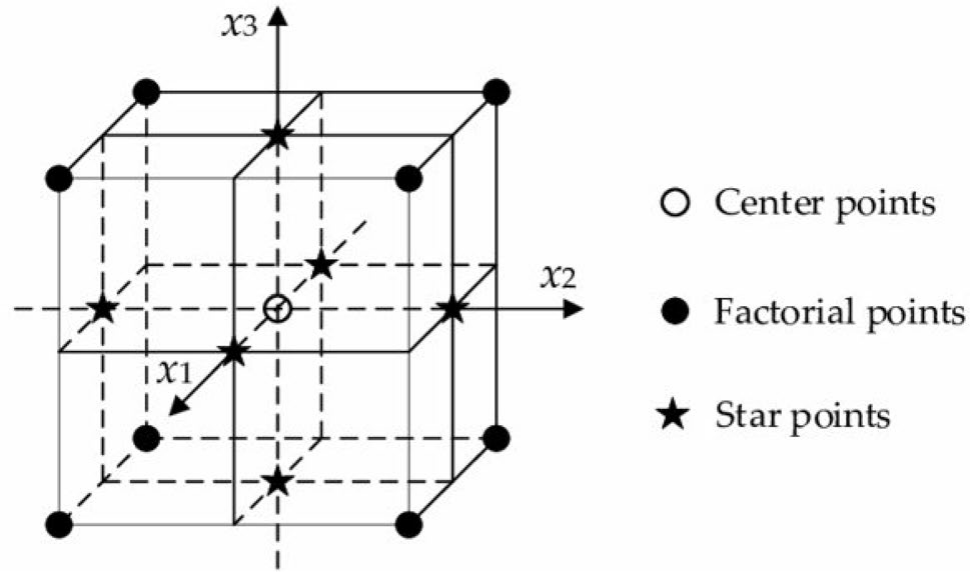
Face-centred central composite design (FCCD) for three factors (*x*_1,_ *x*_2_, *x*_3_) varying in three levels^35^.

Response surface methodology (RSM) is an experimental technique adopted to find the optimal response within specified ranges of the factors. The central composite designs (CCD) are capable of fitting a second order prediction equation for the response. The quadratic terms in prediction equation model the curvature in the true response function. RSM can find a maximum or minimum response within the region of factor space as shown in Fig. 2. A CCD, contains an imbedded factorial or fractional factorial design with centre points that is augmented with a group of ‘star points’ that allow estimation of curvature. If the distance from the centre of the design space to a factorial point is ± 1 unit for each factor, then the distance from the centre of the design space to a star point is |α|> 1. Based on the values of α, the CCD is classified in three categories: (1) circumscribed CCD (α > 1), (2) face-centred CCD (α =  ± 1), (3) Inscribed CCD (α < 1). The precise value of α depends on certain properties desired for the design and on the number of factors involved^32^. The FCCD requires 3 levels of each factor, whereas other two categories of CCD require 5 levels of each factor. The current plan of experiments is varying each factor in 3 levels. The FCCD approach for evaluating the interaction effect of multiple factors as demonstrated by Ahmadi et al.^33^ and Beg et al.^34^ found appropriate for the experimentation.

### Experimental procedure

Experimentation was planned at laboratory settings that replicate the actual manual material handling under consideration. The handling task at the warehouse had variations in terms of slope of the surface (0–10°), speed of walking (0.76–1.21 m/s), footwear (barefoot, sleeper, and shoes), carrying method (CoS, CoB, and CoBp), and load handled (up to 20 kg) as discussed in Section “Activity under consideration”. All these parameters are found to be changing with respect to the participants and the space where the activity is carried out. For the experimentation, five input variables (factors)- (A) footwear type, (B) method of carrying the load, (C) load handled, (D) slope of the walking surface, and (E) speed of walking—were considered. Considering the variations in these factors, three levels of each of them, as shown in Table 2, were set. Design of Experiment (DOE) using RSM was adopted for critically analysing the effect of these five factors on 18 gait parameters (response variables), i.e., (Y_1_) Cadence (steps/min), (Y_2_) Step length (m), (Y_3_), % Double support (DS), (Y_4_) Average gait cycle duration (s), (Y_5_) % Pelvic symmetry, (Y_6_) Pelvic tilt (˚), (Y_7_) Pelvic obliquity (˚), (Y_8_) Pelvic rotation (˚), (Y_9_) Hip flexion -extension (˚), (Y_10_) Hip Abduction- Adduction (˚), (Y_11_) Hip Rotation (˚), (Y_12_) Knee Flexion—Extension (˚), (Y_13_) Knee Abduction- Adduction (˚), (Y_14_) Knee Rotation (˚), (Y_15_) Ankle Dorsi -Plantarflexion (˚), (Y_16_) Ankle Eversion—Inversion (˚), (Y_17_) Ankle Rotation (˚), and (Y_18_) Foot tilt Vs Horizontal (˚). Out of these 18 parameters, which collectively represent lower extremity kinematics, five parameters (i.e., Y_1_ to Y_5_) indicate spatiotemporal gait parameters and remaining 13 parameters (i.e., Y_6_ to Y_18_) indicate joint angles in the lower extremity. Figure A.1 (Appendix) shows the typical spatio-temporal gait parameters as recorded for subject 12. Figure A.2 shows 13 lower extremity joint angles, i.e., Y_6_ to Y_18_.

**Table 2:** Experimental ranges of levels of the five factors.

Sr. no	Factor	Range of levels		
		Level 1	Level 2	Level 3
1	Footwear type (A)	Barefoot	Sleepers	Shoes
2	Method of carrying (B)	Carrying on back (CoB)	Carrying on shoulder (CoS)	Carrying on backpack (CoBp)
3	Load handled (C)	0 kg	10 kg	20 kg
4	Slope of walking surface (D)	0°	5°	10°
5	Speed of walking (E)	0.76 m/s (1.7 miles/hr)	0.98 m/s (2.2 miles/hr)	1.21 m/s (2.7 miles/hr)

The RSM-FCCD yielded 28 trial runs as shown in Table 3. In addition to these 28 experimental runs, initial baseline trial (0^th^ run) was carried out for each subject to understand the subject’s normal gait. Thus, total experimentation trial runs were 29. For baseline trial, the subjects were asked to walk barefoot on level ground without carrying any load at a self-paced walking speed. Experimental runs were carried out after the subjects got comfortable for treadmill walking at variable speeds, slopes and loads.

**Table 3:** Design of experiment table showing the levels of input parameters.

Run no	(A) Footwear	(B) Carrying method	(C) Load handled	(D) Slope of surface	(E) Walking speed
0	Baseline run for normal gait (barefoot, without load, level ground, and self-selected speed)				
1	2	2	2	2	2
2	2	2	2	2	2
3	3	3	3	3	1
4	3	1	3	1	1
5	3	3	1	3	3
6	2	2	2	2	3
7	2	2	2	1	2
8	2	2	2	3	2
9	1	3	1	3	1
10	1	3	3	3	3
11	1	3	3	1	1
12	3	1	3	3	3
13	1	2	2	2	2
14	3	2	2	2	2
15	2	2	1	2	2
16	3	3	1	1	1
17	1	1	1	1	1
18	2	3	2	2	2
19	1	1	3	3	1
20	2	2	2	2	1
21	2	2	3	2	2
22	3	1	1	1	3
23	3	3	3	1	3
24	1	1	1	3	3
25	3	1	1	3	1
26	1	3	1	1	3
27	2	1	2	2	2
28	1	1	3	1	3

### Data collection

The lower extremity kinematics, i.e., 18 gait parameters were captured using sensors-based gait measurement system (iSen model, STT systems, San Sebastián, Spain). iSen provides specific protocol for placing the sensors on body landmarks. Though iSen assumes that the transformation between the sensor local reference frame and the body segment reference frame remains constant throughout the recording, we adopted the following guidelines provided by the Original Equipment Manufacturer (OEM). (i) Choose a landmark such that the relative motion between sensor and bone is minimized. (ii) The landmark to be chosen on –(a) flat, harder regions, (b) regions with a smaller muscular mass, and (c) regions where skin-folds throughout a joint range of motion have a smaller impact.

**Figure 3:**
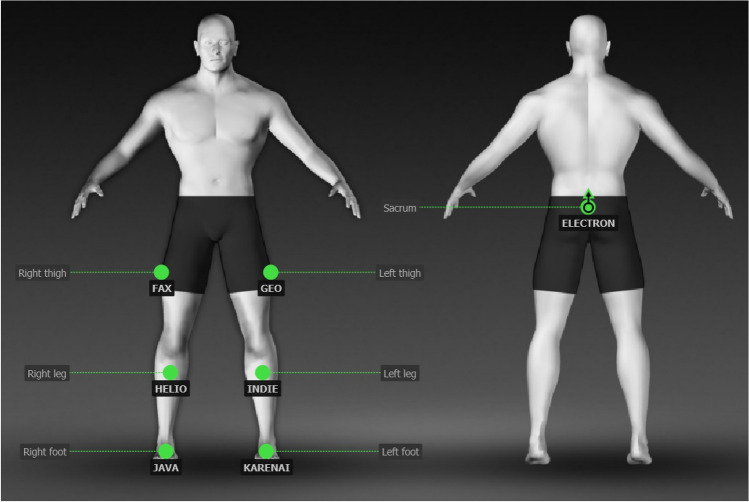
Inertial sensor placement on body regions.

The seven inertial sensors, ELECTRON, FAX, GEO, HELIO, INDIE, JAVA and KARENAI, were mounted respectively on the body landmarks—sacrum, right thigh, left thigh, right leg, left leg, right foot and left foot, using straps as per the lower body gait protocol specified for iSen software. The sensor placement as per the protocol^36,37^ is depicted in Fig. 3.

iSen software provides the provision for global calibration, which ensures that all the sensors are set at the proper positions. For global calibration, iSen uses a global reference frame which is an external reference frame common to all sensors. It is typically determined by the vertical axis (accelerometer output while sensors are still) and a certain heading. During the experiment, the IMU sensors were arranged with arrow head pointing vertically upward. Because of the movement of subjects, drifts in the sensors are likely to take place. This drift was detected and eliminated by two ways- (i) by performing global calibration at the start of each trial, and (ii) by comparing the silhouette video (which is obtained from the sensor data) with normal video of walking. The iSen software has capability to identify the initial support and toe-off events in the recording which determines the gait cycle. The accuracy of iSen system is among the best of its kind: Pitch/roll < 0.5 degrees (Root Mean Square (RMS)) and Heading < 2.0 degrees (RMS).

### Data processing

The data regarding lower extremity kinematics was collected for 15 subjects, each executing 29 trials, using iSen system. The iSen software presents the silhouette motion generated from sensor data and plays the video of motion of subject. Each trial was performed repeatedly by comparing the silhouette motion with actual video of trial to minimize the error. The complete recording comprises of calibration frame, initial walking, rhythmic gait and terminal gait for approximately 2 min. The rhythmic gait data of 30 s approximately were extracted from the complete recording for evaluation. In between the trials, subjects were allowed to take sufficient rest. For each of 435 trial executions, data of five spatio-temporal parameters and ranges of motion (ROM) for 13 joint angles in the lower extremity were recorded. Table 4 depicts the sample data for 18 gait parameters for subject 12. A statistical software JMP (v5.1) was used to analyse the data collected through 435 trial executions.

**Table 4:** Response variable data for 29 trials (for subject 12).

Response variable ↓	Run no																												
	0	1	2	3	4	5	6	7	8	9	10	11	12	13	14	15	16	17	18	19	20	21	22	23	24	25	26	27	28
(Y_1_) Cadence (steps/min)	113	100	100	96.7	96.5	109	112	104	103	101	129	108	117	110	104	110	98.4	111	107	100	94.1	106	111	114	131	87.3	124	101	119
(Y_2_) Step length (m)	0.3	0.3	0.3	0.4	0.4	0.3	0.3	0.4	0.4	0.4	0.3	0.3	0.3	0.3	0.4	0.3	0.4	0.3	0.3	0.4	0.4	0.3	0.3	0.3	0.3	0.4	0.3	0.4	0.3
(Y_3_) % Double support	11	9.2	9.2	6.9	12.7	7.4	8	11.3	7.9	8.9	8	7.3	8	8.4	7.9	8.1	8.8	8.3	7.1	7.3	8.7	9.3	11.6	13.2	8.3	9.2	9.6	10.9	9.8
(Y_4_) Avg gait cycle duration (s)	1	1.1	1.1	1.2	1.2	1	1	1.1	1.1	1.2	0.9	1.1	1	1	1.1	1	1.2	1	1.1	1.1	1.2	1.1	1	1	0.9	1.3	0.9	1.1	1
(Y_5_) % Pelvic symmetry	41	48	48	37	31	40	47	44	45	40	36	42	41	40	47	51	39	36	43	36	46	43	48	38	45	40	42	40	39
(Y_6_) Pelvic tilt (˚)	9	11	11	8	14	9	13	11	12	13	11	11	11	10	11	9	9	12	9	10	10	11	8	12	11	12	11	9	10
(Y_7_) Pelvic obliquity (˚)	8	10	10	12	8	7	11	11	7	7	5	4	8	7	8	8	7	10	5	10	9	8	12	10	9	6	10	7	6
(Y_8_) Pelvic rotation (˚)	23	12	12	10	11	14	17	19	16	16	14	11	14	12	21	14	13	26	13	16	21	13	17	12	17	21	18	13	10
(Y_9_) Hip flexion -extension (˚)	41	58	58	61	47	67	64	54	66	60	71	44	68	53	57	49	50	47	57	58	51	55	57	64	62	60	51	52	51
(Y_10_) Hip Abduction- Adduction (˚)	19	22	22	16	18	19	21	24	17	14	18	13	22	15	21	16	20	20	15	16	19	20	24	23	17	15	22	18	21
(Y_11_) Hip Rotation (˚)	17	29	29	25	20	31	26	22	31	35	26	22	33	24	26	23	21	15	27	32	29	24	23	27	28	31	25	28	27
(Y_12_) Knee Flexion- Extension (˚)	67	70	70	74	74	69	72	81	72	64	66	61	69	65	75	69	80	74	71	61	76	74	79	78	63	73	72	66	70
(Y_13_) Knee abduction–adduction (˚)	9	21	21	22	19	21	21	19	24	21	26	15	26	15	20	15	17	13	20	22	18	16	21	26	20	20	24	17	17
(Y_14_) Knee rotation (˚)	22	34	34	26	30	29	32	31	37	35	29	26	29	28	30	26	27	24	34	30	31	27	32	31	34	33	43	31	31
(Y_15_) Ankle dorsi-plantarflexion (˚)	26	43	43	34	24	37	47	36	45	42	56	32	37	45	37	36	26	32	35	45	44	35	31	42	45	32	37	43	49
(Y_16_) Ankle eversion—inversion (˚)	21	25	25	20	16	17	27	35	32	26	28	23	16	24	22	21	24	26	32	25	28	25	18	17	22	25	26	28	22
(Y_17_) Ankle rotation (˚)	19	16	16	22	21	24	25	25	24	27	38	22	30	29	24	19	22	23	20	32	26	23	23	26	23	26	21	22	24
(Y_18_) Foot tilt vs horizontal (˚)	64	97	97	80	78	101	106	92	101	83	89	67	86	88	96	88	84	68	86	79	89	91	101	108	90	92	87	99	95

### Statistical analysis

The least square fit regression model was applied to the data of each subject, i.e., input values of factors (Table 3) and the corresponding values of the response variables obtained (Table 4). A second order polynomial equation was obtained by fitting the data using multiple regression process. This generated an empirical model which relates the response measured (Y_i_) to the independent variables (A, B, C, D, and E) in the experiment. For a five-factor system, the Eq. (1) shows generalized regression model.

1 $${\mathbf{Y}}_{{\mathbf{i}}} = \beta_{{0\left( {\text{i}} \right)}} + \beta_{{{1}\left( {\text{i}} \right)}} {\text{A }} + \beta_{{{2}\left( {\text{i}} \right)}} {\text{B }} + \beta_{{{3}\left( {\text{i}} \right)}} {\text{C }} + \beta_{{{4}\left( {\text{i}} \right)}} {\text{D }} + \beta_{{{5}\left( {\text{i}} \right)}} {\text{E }} + \beta_{{{11}\left( {\text{i}} \right)}} {\text{A}}^{{2}} + \beta_{{{22}\left( {\text{i}} \right)}} {\text{B}}^{{2}} + \beta_{{{33}\left( {\text{i}} \right)}} {\text{C}}^{{2}} + \beta_{{{44}\left( {\text{i}} \right)}} {\text{D}}^{{2}} + \beta_{{{55}\left( {\text{i}} \right)}} {\text{E}}^{{2}} + \beta_{{{12}\left( {\text{i}} \right)}} {\text{AB }} + \beta_{{{13}\left( {\text{i}} \right)}} {\text{AC }} + \beta_{{{14}\left( {\text{i}} \right)}} {\text{AD }} + \beta_{{{15}\left( {\text{i}} \right)}} {\text{AE }} + \beta_{{{23}\left( {\text{i}} \right)}} {\text{BC }} + \beta_{{{24}\left( {\text{i}} \right)}} {\text{BD }} + \beta_{{{25}\left( {\text{i}} \right)}} {\text{BE }} + \beta_{{{34}\left( {\text{i}} \right)}} {\text{CD }} + \beta_{{{35}\left( {\text{i}} \right)}} {\text{CE }} + \beta_{{{45}\left( {\text{i}} \right)}} {\text{DE}},$$

where, Y_i_ is predicted response (Y_1,_ Y_2,_ ……., Y_18_), β_0(i)_ is an intercept, β_1(i)_, β_2(i)_, β_3(i)_, β_4(i)_, β_5(i)_ are linear coefficients, β_11(i)_, β_22(i)_, β_33(i)_, β_44(i)_, β_55(i)_ are squared coefficients, β_12(i)_, β_13(i)_, β_14(i)_, β_15(i)_, β_23(i)_, β_24(i)_, β_25(i)_, β_34(i)_, β_35(i)_, β_45(i)_ are interaction coefficients.

The RSM approach involves fitting the quadratic model, evaluating the values of coefficients, ANOVA, parameter estimates and building plots which presents interactions effects between variables and responses.

### Statement

All the authors confirms that the methods were carried out in accordance with relevant national/international regulations and guidelines.

All the authors confirms that all experimental protocols were approved by Visvesvaraya National Institute of Technology, Nagpur, Maharashtra, India.

All the authors confirms that the informed consent was obtained from all subjects and/or their legal guardian(s).

## Results and discussions

A significant amount of useful data has been generated by performing a gait analysis of 15 participants in 29 trials. The factor combinations in the trials covers all the major variations in manual material handling tasks under consideration. Further, these trials include possible intervention strategy, i.e., use of backpack. The least-square (quadratic) model was fitted to the data shown in Table 4 to identify the individual factor effects and two-factor interactions. The analysis tests the hypotheses as follows:

H_0_: Change in response variable is independent of change in input factors, i.e., change is due to chance cause. (β_0(i) =_ β_1(i) =_ β_2(i) ………_ β_45(i)_ = 0)

H_1_: Change in response variable is dependent on change in input factors. (i.e., at least one β is non zero)

As evident from Table 5, for majority of subjects, 14 gait parameters (out of 18) show significance of p ≤ 0.05. It suggests that the changes in these gait parameters are not by chance causes but due to changes in the input variables (βs ≠ 0), thereby, rejecting the null hypothesis. The remaining four parameters, such as pelvic symmetry % (Y_5_), pelvic rotation (Y_8_), knee rotation (Y_14_), and ankle rotation (Y_17_) are found to be statistically insignificant (p > 0.05) for majority (more than 7) of subjects. This means that the changes in these four gait parameters are due to chance cause and not due to change in input factors. Thus, Y_5_, Y_8_, Y_14_ and Y_17_ were eliminated and the further assessment is carried for 14 significant gait parameters.

**Table 5:** Significance analysis of response variables for the 15 subjects.

Sr. no	Responses variable (Y)	No. of subjects with p value > 0.05	No. of subjects with p value < = 0.05
1	Cadence (steps/min) (Y_1_)	1	14
2	Step length (m) (Y_2_)	7	8
3	% Double support (Y_3_)	4	11
4	Average gait cycle duration (Sec) (Y_4_)	5	10
5	Pelvic symmetry % (Y_5_)	12	3
6	Pelvic tilt (Degrees) (Y_6_)	5	10
7	Pelvic obliquity (Degrees) (Y_7_)	5	10
8	Pelvic rotation (Degrees) (Y_8_)	9	6
9	Hip flexion -extension (Degrees) (Y_9_)	0	15
10	Hip abduction—adduction (Degrees) (Y_10_)	6	9
11	Hip rotation (Degrees) (Y_11_)	4	11
12	Knee flexion—extension (Degrees) (Y_12_)	1	14
13	Knee abduction—adduction (Degrees) (Y_13_)	5	10
14	Knee rotation (Degrees) (Y_14_)	9	6
15	Ankle dorsiflexion—plantarflexion (Degrees) (Y_15_)	3	12
16	Ankle eversion -inversion (Degrees) (Y_16_)	4	11
17	Ankle rotation (Degrees) (Y_17_)	9	6
18	Foot tilt Vs horizontal (Degrees) (Y_18_)	3	12

Change in a factor level may create one of the five possible effects on a response- (i) increase, (ii) decrease, (iii) increase followed by decrease, (iv) decrease followed by increase, (v) no significant change. However, as evident from the experimental results, different subjects revealed different trends of the responses with respect to the change in factor. For example, 13 subjects revealed decreasing trend in cadence as footwear level was changed from 1 to 3 (i.e., barefoot to sleeper to shoes). Similarly, in case of 14 subjects, cadence was found to be increasing with respect to increase in the level of speed from 1 to 3 (i.e., 0.76 m/s to 0.98 m/s to 1.21 m/s). The effects observed in the case of majority of subjects were considered prominent and are depicted in Table 6. The cells highlighted in green represents the prominent effect of factor on gait parameter.

**Table 6:**
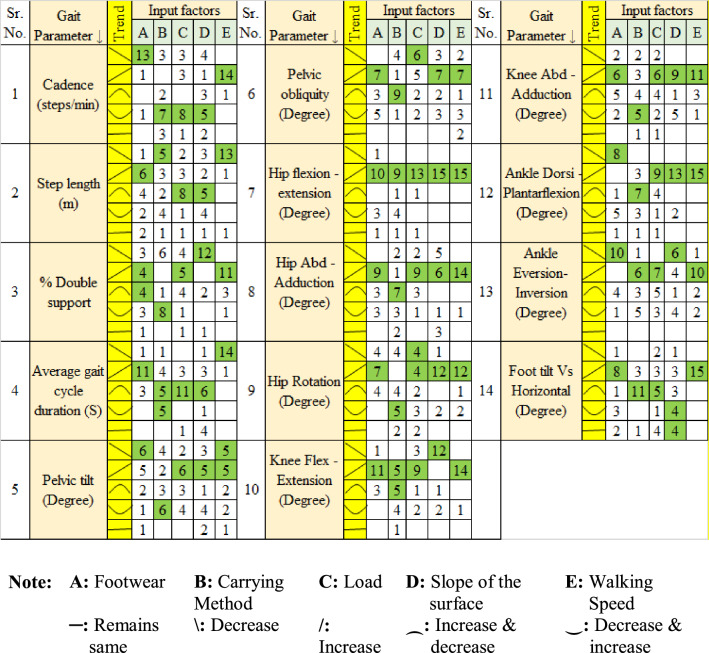
Number of subjects revealing different patterns of input–output relationship.

1. As the speed increases from 0.76 m/s to 1.21 m/s, cadence, % DS, pelvic obliquity angle, hip angles, knee angles, and ankle angles increases; whereas step length and average gait cycle duration decreases.
2. As the load increases from 0 to 20 kg, % DS, pelvic angles, hip angles, knee angles, and ankle angles increases; whereas mixed responses are observed for cadence, step length, and average gait cycle duration.
3. As the slope of walking surface increases from 0 to 10°, pelvic angles, hip angles, knee adduction-abduction angle, ankle plantar-dorsiflexion angle increase; knee flexion–extension angle, ankle eversion-inversion angle, and % DS decreases; mixed responses are observed for other gait parameters.
4. As the footwear varies from barefoot to sleeper to shoes, knee angles, hip angles, pelvic obliquity angle, % DS, step length, and average gait cycle duration increases. In contrast, cadence, pelvic tilt angle, and ankle angles decrease.
5. As the carrying method varies from CoB to CoS to CoBp, cadence, % DS, pelvic tilt, hip rotation angle, and knee adduction-abduction angle decrease initially and then increase. In contrast, step length decreases. Ankle eversion-inversion angle, hip flexion–extension angle increases, pelvic obliquity angle, hip adduction-abduction angle, and ankle plantar-dorsiflexion angle increase initially and then decrease.
6. Hip flexion–extension increased with respect to rise in the level of all five factors, whereas hip adduction-abduction, hip rotation, knee flexion–extension and ankle plantar-dorsiflexion shows increasing behaviour with respect to rise in three factors. We can summarise that, hip joint angles and flexion–extension angles of lower extremity increases with rise in level of most of the factors.

It should be noted that the trends depicted in Table 6 are based on single-factor effect. It would be interesting to explore the two-factor interaction effects on the gait parameters. The combined effect of two or more factors on the gait is significant and different from that of a single factor.

The relative contribution of each factor (A to E) to each dependent variable (Y_1_ to Y_18_) was directly measured by the respective coefficient in the fitted model (Eq. (1)). A positive sign for the coefficients (β’s) in the fitted models for Y_i_ indicated that the level of the gait parameter Y_i_ increased with increase in the level of corresponding factor. Among the 4410 (= 21 coefficients × 14 parameters × 15 subjects) coefficients, the largest coefficient (β_5_ = 11.25) was observed for cadence (Y_1_) of subject 05, which revealed the high sensitivity of cadence to walking speed for that subject. On the other hand, the lowest coefficient (β_15_ = 0.019) was obtained for cadence (Y_1_) of subject 09 which indicated that the combined effect of load handled and carrying method was less prominent on the cadence for the subject. Moreover, a negative sign for the regression coefficient of β_1_ for subject 10 indicated that the cadence decreased as the footwear level decreased. The signs of coefficients (positive or negative) were observed for all 14 responses and 15 subjects, and based of majority of signs, the positive or negative trends were decided. If distinct majority (of either positive or negative signs) was missing, then trends were labelled as mixed trends. The results thus obtained are encapsulated in Table 7. A few of the key findings obtained after analysing single-factor and two-factor interactions in a way discussed above are presented below.

1. The speed of walking is found to be dominant factor affecting the gait parameters. The results showed that the regression coefficient β_5_ (of walking speed, E) for step length (Y_2_), average gait cycle duration (Y_4_), and pelvis tilt (Y_6_) have negative signs. Thus, for the given range of walking speed, the increase in walking speed will decrease the step length, average gait cycle duration, and pelvis tilt. The positive sign for coefficients other than these three parameters signifies that the increase in walking speed increases the values of these parameters. These effects of walking speed are based on parameter estimates (i.e., signs of regression coefficients), which are coherent with the single-factor effect portrayed in Table 6.
2. Like single factor effect, the effect of squared interactions and two-factor interactions on various gait parameters can be positive, negative or mixed. Table 7 highlights the behaviour of gait parameters subjected to the combinations of input factors based on majority rule discussed earlier. The Table 7 shows that the combined effect of two factors is different than that of individual effect. For example, the change in AB level (footwear type and carrying method) increases the cadence (Y_1_). Individual variation in the level of A (footwear type) decreases the cadence (Y_1_) and variation in B (carrying method) has mixed responses for different subjects. Also, combined variation of AB (footwear and carrying method) resulted in increasing pelvic obliquity (Y_7_), hip flexion–extension (Y_9_), hip adduction-abduction (Y_10_), knee flexion–extension (Y_12_), knee adduction-abduction (Y_13_), and ankle dorsiflexion-plantarflexion (Y_15_). However, combined variation of AB (footwear and carrying method) decreased % double support (Y_3_), average gait cycle duration (Y_4_), hip rotation (Y_11_), and foot tilt vs horizontal (Y_18_).

**Table 7:** Single factor and two-factor interaction effect on gait parameters observed on the study population.

Coefficient (Interaction) ↓	Gait parameter (Y_i_)													
	Y_1_	Y_2_	Y_3_	Y_4_	Y_6_	Y_7_	Y_9_	Y_10_	Y_11_	Y_12_	Y_13_	Y_15_	Y_16_	Y_18_
β0 (Intercept)	P	P	P	P	P	P	P	P	P	P	P	P	P	P
β_1_ (A)	N	P	M	P	M	P	P	P	M	P	P	N	N	P
β_2_ (B)	M	N	N	P	M	N	P	M	N	P	P	P	P	P
β_3_ (C)	M	P	P	M	P	M	P	P	P	P	P	P	P	M
β_4_ (D)	M	N	N	N	M	P	P	M	P	N	P	P	P	P
β_5_ (E)	P	N	P	N	N	P	P	P	P	P	P	P	M	P
β_11_ (A*A)	N	P	N	N	M	M	M	M	P	M	N	N	N	P
β_22_ (B*B)	P	N	M	N	M	M	N	P	N	N	M	N	N	N
β_33_ (C*C)	M	N	N	N	N	M	P	N	N	P	M	P	N	P
β_44_ (D*D)	M	P	M	N	M	M	N	N	N	N	N	N	N	N
β_55_ (E*E)	M	N	P	N	N	P	P	M	N	P	N	P	M	N
β_12_ (AB)	P	N	N	N	M	P	P	P	N	P	P	P	M	N
β_13_ (AC)	M	N	N	N	N	P	M	P	M	N	M	N	N	N
β_14_ (AD)	N	P	N	M	M	P	M	P	P	M	M	M	M	P
β_15_ (AE)	M	N	N	M	M	N	N	N	M	N	N	M	M	M
β_23_ (BC)	P	N	N	N	P	N	N	N	N	N	N	N	M	N
β_24_ (BD)	P	N	N	M	P	M	N	N	N	N	N	P	M	M
β_25_ (BE)	P	N	P	M	P	N	P	N	M	M	M	N	P	N
β_34_ (CD)	P	N	N	N	P	M	N	N	P	M	M	M	N	N
β_35_ (CE)	P	N	M	N	M	P	P	P	M	P	P	P	P	M
β_45_ (DE)	N	N	M	P	P	P	M	P	P	N	M	M	P	P

Individual effect: A, B, C, D, E; Squared interaction: A^2^, B^2^, C^2^, D^2^, E^2^.

Two-factor interaction: AB, AC, AD, AE, BC, BD, BE, CD, CE, DE.

*P* Positive effect, *N* Negative effect, *M* Mixed effect.

**Figure 4:**
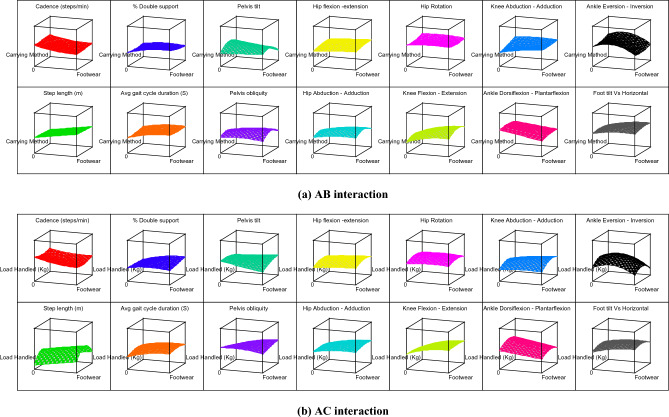

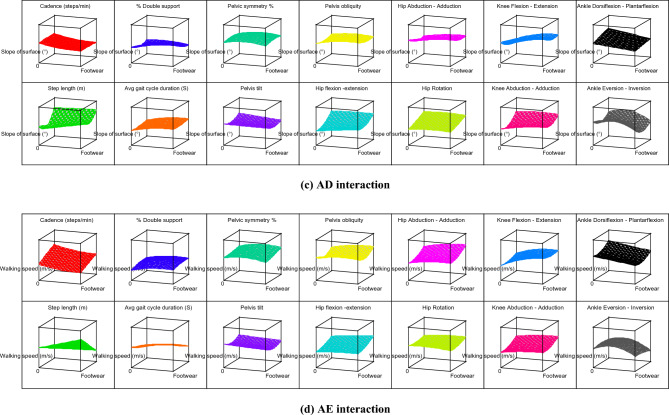

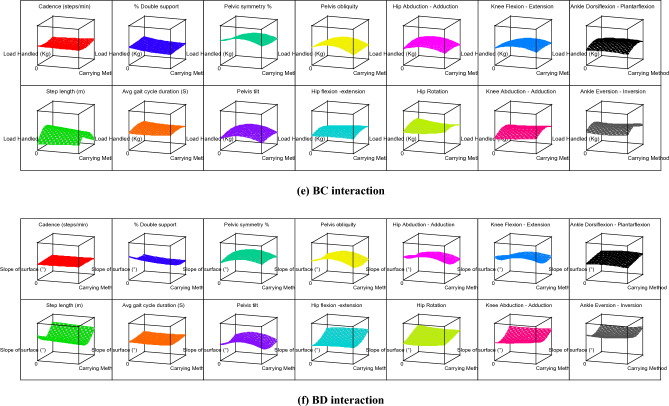

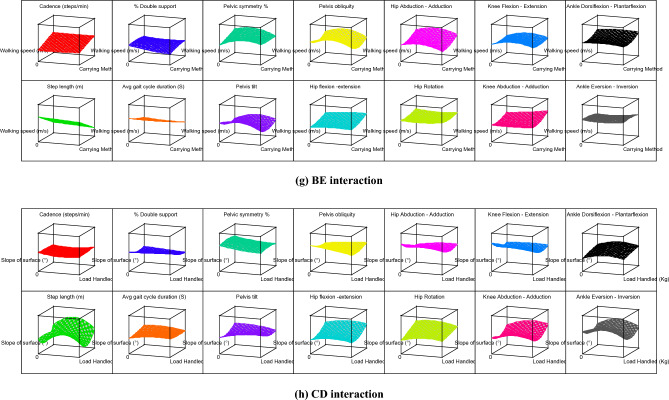

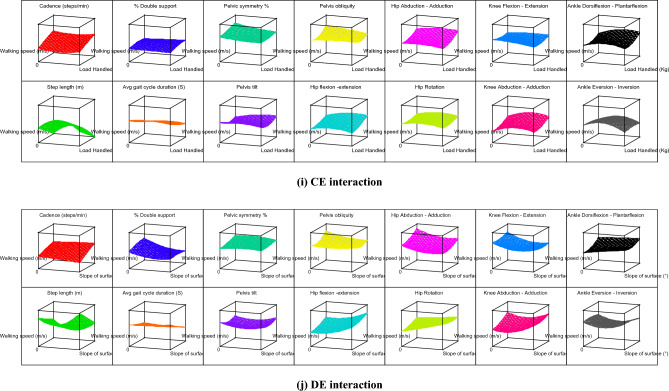
Contour graphs showing effect of two-factor interaction on 14 gait parameters for subject 12.

**Figure 5:**
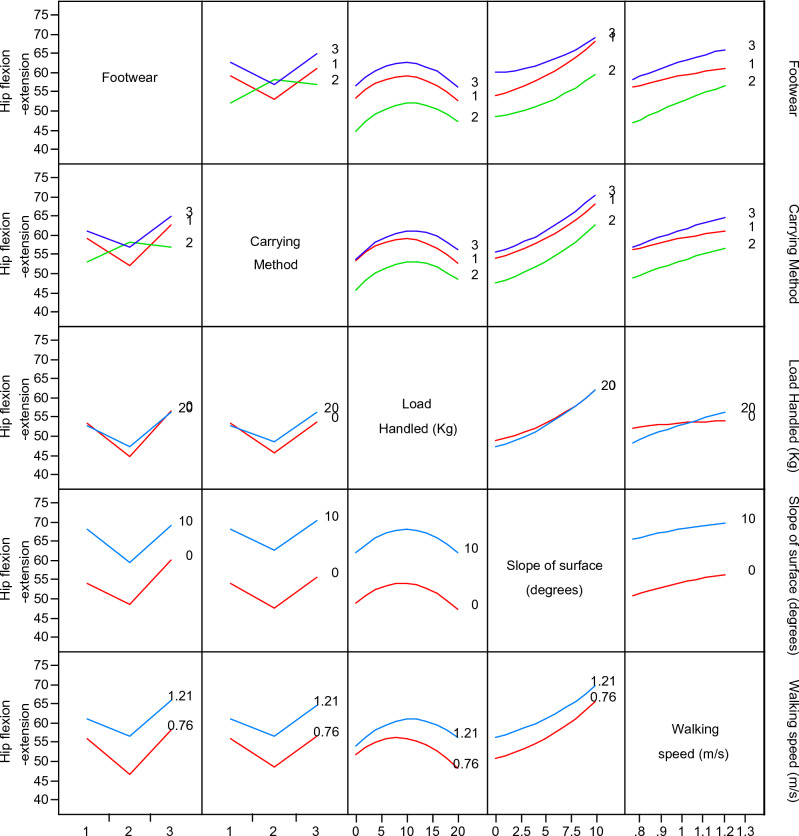
Effect of two-factor interaction on hip flexion–extension for subject 12.

The contour graphs depicting the effect of 10 two-factor interactions, such as AB, AC, AD, AE, BC, BD, BE, CD, CE, and DE on the 14 gait parameters for subject 12 are shown in Fig. 4. For better understanding, effect of two-factor interactions on hip flexion–extension (Y_9_) for subject 12 is dpicted in Fig. 5. The graph in Fig. 5 shows three lines for ordinal factors (footwear and carrying method) and two lines for continous factors (Speed, slope and load). In general, for all two-factor interactions, hip flexion–extension ROMs were found to be increasing with higher level of factors, such as shoes (footwear), carrying on backpack, walking surface slope of 10° and walking speed of 1.21 m/s. It should be noted that lower hip flexion–extension ROM (in case of carrying methods) indicates restriction for hip movement which is prominently observed for CoB & CoS. On the other hand, incerased ROM of hip flexion–extension for higher slope of walking surface indicates additional compensation for balancing. Both this conditions, i.e., restriction for hip movement and compensation for balancing are likely to create stresses in lower extremity. Thus, use of backpack and lower slope of walking surface are highly recommneded for the manual material handling under consideration.

It is observed from Table 7, that as the load handled (C) increases, ROMs of majority of joint angles increase, such as flexion–extension/ adduction-abduction/ eversion-inversion/ rotation angle of lower extremity (i.e., Y_6_ & Y_9_ to Y_16_ respectively). These ROMs are more than that of normal walking (i.e., 0^th^ trial). These findings match with that of Dames & Smith^27^. The frequent excessive joint ROMs lead to bending the trunk laterally, posteriorly, and anteriorly. This is one of the risk factors leading to painful hip, hip abductor (muscles) weakness, inadequacy of the knee extensors, hip extensor weakness, hip flexion contracture, hip ankylosis (fused), and ankle pain. These discomforts are the possible symptoms of gait deformities, such as lateral trunk bending/ipsilateral lean/Trendelenburg gait, ankylosis, and waddling^17^. The interaction effects of footwear and load (AC), and carrying method and load (BC), are negative for many response variables. On the other hand, interaction effect of load and slope (CD), is negative for fewer gait parameters. Many gait parameters were found to have positive effect of the interaction of load and speed (CE). The inference drawn based on these interactions supports reduction in the load handled, use of shoes, use of backpack and walking along moderate slope or level ground with self-paced speed. These suggestions will help to design the safe handling tasks.

It is observed that variations in most of the parameters (except step length, average gait cycle duration, and pelvic tilt) are directly proportional to walking speed. These findings are coherent with that of Bovi et al.^38^. During self-paced walking, the speed of walking for an individual is usually constant. The applied load, abnormal posture acquired through the method of loading, footwear, and slope of the surface may affect the walking speed. The speed of walking is responsible for increasing the load on legs and developing stresses in the lower extremity muscles and joints^22,39^. The effect of interaction of footwear and speed (AE) is negative on all gait parameters. It suggests that shoes and moderate walking speeds are the best intervention to optimize the gait parameters. The load and speed interaction (CE), and slope and speed interaction (DE), show positive effect on the parameters.

As discussed earlier, the carrying method has been an important factor that affects the gait pattern. The unbalanced load held in the body's front, side, and back disturbs the body's equilibrium and balance^23^. The footwear and carrying method (AB) interaction shows that the CoS and barefoot combination results in elevated values for most of the gait parameters. Thus, CoS & barefoot walking simultaneously indicates hazardous combination. The CoBp and use of shoes is best out of other combinations in AB interaction. It is further observed that level 2 of speed, slope, and load (i.e., 0.98 m/s, 5^°^, and 10 kg respectively) in various interactions results in the rise of response variables. Similarly, other interactions can be compared by observing Table 7, Figs. 4 and 5.

In the activity of carrying the grain bags, the workers adopt specific postures and the risk associated with these postures is evaluated by Adhaye and Jolhe^40^. Obviously, these postures are due to different settings of the five task characteristics. Findings of the present study (which essentially considers the same activities as in Ref.^40^) further supports the fact that postures are riskier and leads to MSDs in longer term.

All the above interpretations are based on behaviours of each parameter for the 15 subjects within specified age group. However, in some cases, mixed responses of individual factors and two-factor interactions were observed, which are most likely to be due to varying body mass index of subjects. This highlights the scope for further research to identify the causes of mixed interaction and eliminate them.

## Conclusion

The risk involved in the activity of manual carrying of gain bags is significant, often leading to musculoskeletal disorders among the workers. This risk needs to be critically analyzed by adopting some rational technique such as gait analysis. The present research adopts sensor-based gait measurement approach to explore the effects of task characteristics on the gait parameters of concern. The five task characteristics, i.e., footwear type, method of carrying, load handled, slope of walking surface, and speed of walking, were varied at three levels and the effect of each on the 18 gait parameters of 15 subjects were studied, by adopting RSM-FCCD approach. The experimental trials enabled to understand the effects of single factor and two-factor interactions which revealed interesting facts. The two-factor interaction effects are found to be different from the effects of respective individual factor.

For majority of the participants, 14 gait parameters were found to be affected by one or more task characteristics. It is observed that the speed of walking and the load handled are the major factors that affect the gait parameters. Increase in load handled increases ROMs of the joint angles which results in bending of the trunk in different planes ultimately leading to disorders like hip pain, ankle pain, muscles weakness, hip ankylosis, etc. The effect was found to be minimised by employing specially devised a backpack for carrying the load. The postures resulted from the various combinations of task characteristics were observed as sources for developing musculoskeletal disorders in workers. Lower hip flexion–extension ROM, observed in CoB & CoS, highlights restriction for hip movement. Whereas, higher ROM of hip flexion–extension for larger slope of walking surface indicates additional compensation for balancing. Both this conditions are likely to create stresses in lower extremity. Thus, use of backpack and lower slope of walking surface are highly recommneded for the manual material handling under consideration. In general, walking speed of 0.98 m/s, slope of surface of 5°, load of 10 kg, shoes as footwear, and carrying on backpack are found to be optimal combination to minimise the risk. It would be interesting to explore the role of backpack in correcting the postures by carrying out stability assessment study.

### Supplementary Information

Supplementary Figures.

## Data Availability

The datasets used and/or analyzed during the current study available from the corresponding author on reasonable request.
