# Supplementary material for: Biomechanical investigation of tasks concerning manual materials handling using response surface methodology
Source: Sci Rep. 2023 Oct 4;13:16718. doi: 10.1038/s41598-023-43645-2 (PMC10550981; doi:10.1038/s41598-023-43645-2)
Supplement: Supplementary file 1 — Supplementary Figures. [file 41598_2023_43645_MOESM1_ESM.docx]

**Appendix: Gait parameters (Y_1_ to Y_18_)**

(* values indicated are for subject 12)

| **Gait Parameters** | | | | | | | | | | | | |
| --- | --- | --- | --- | --- | --- | --- | --- | --- | --- | --- | --- | --- |
| **(Y_1_) Average Cadence:** | | | | |  | | | **(Y_5_) Pelvic Symmetry:** | | | |  |
| 96.5 steps/min * | | | | |  | | | 31 % * | | | |  |
| 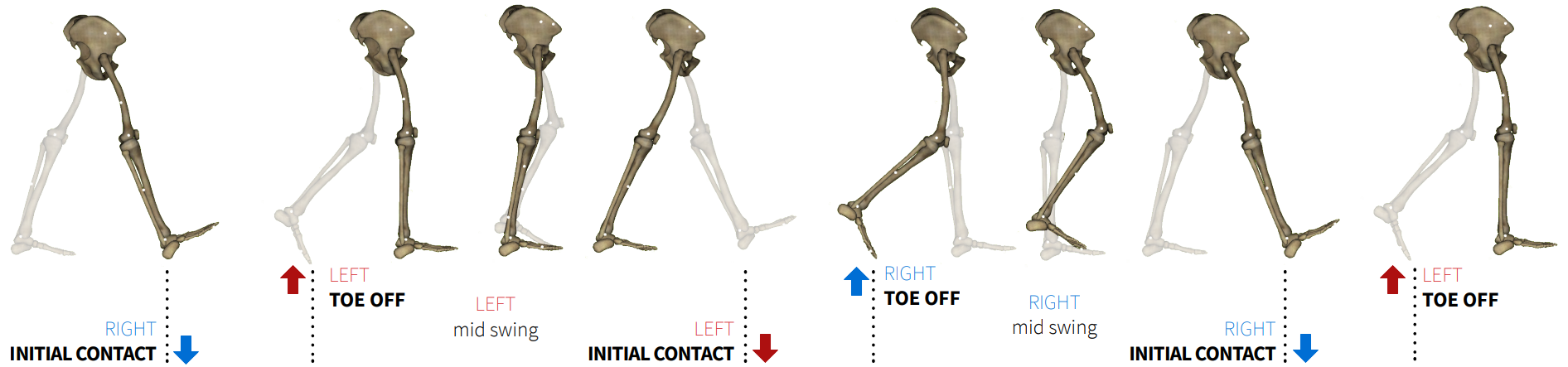 | | | | | | | | | | | | |
| **0%** | |  |  |  | |  | |  | **100%** | |  | |
|  | **Right Stance Phase:**  **0.6 sec** (52.9 %) | | | | | | **Right Swing Phase:**  **0.5 sec** (47.0 %) | | |  |  | |
|  | **Double support** |  |  |  | | **Double support** |  |  |  | **Double support** | **(Y_3_) Double Support: 6.9 % *** | |
|  |  | **Left Swing Phase:**  **0.5 sec** (45.9 %) | | | | **Left Stance Phase:**  **0.6 sec** (54.0 %) | | | | |  | |
|  | **0%** | |  |  | | |  |  |  | **100%** | | |
| **(Y_4_) Average Gait Cycle Duration:** 1.2 sec * | | | | | | | | | | | | |

|  |  | | **Left Step Length:** 0.3 m | |  | |
| --- | --- | --- | --- | --- | --- | --- |
|  |  | |  | |  | |
| 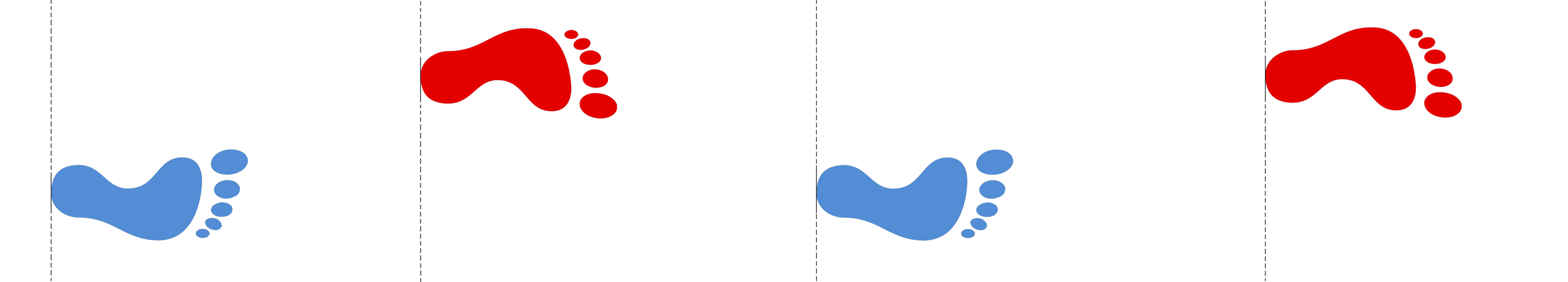 | | 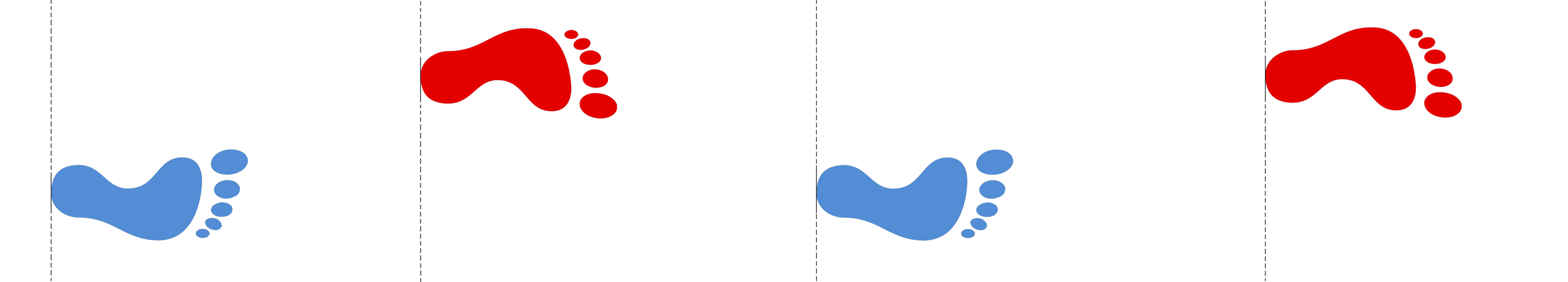 | | 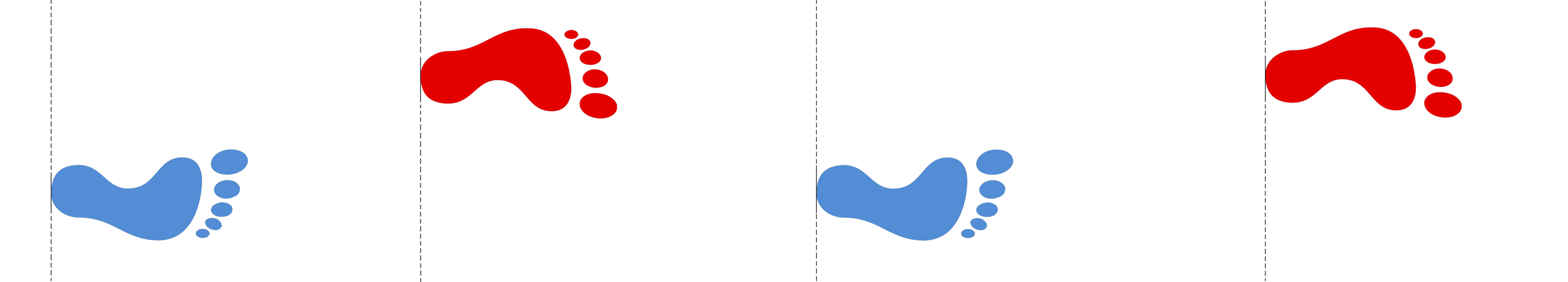 | |  |
|  | **Right Step Length:** 0.4 m | |  | | **(Y_2_) Average Step Length:** 0.4 m * | |
|  | **Right Stride Length: 0.7 m** | |  | | **(**Average of 20 cycle) | |
|  |  | |  | |  | |

**Figure A.1:** Spatio-temporal gait parameters Y_1_ to Y_5_ *

| **PELVIC** | **HIP** | **KNEE** | **ANKLE** |
| --- | --- | --- | --- |
| 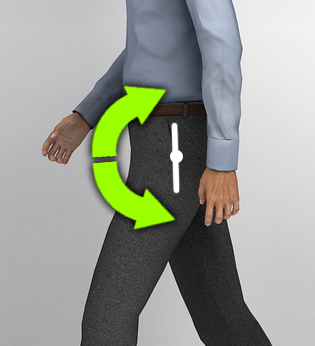 | 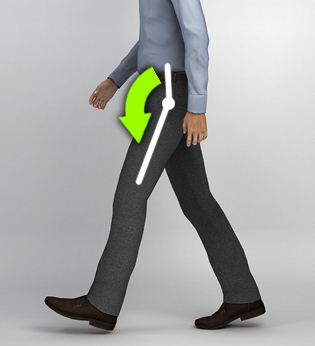 | 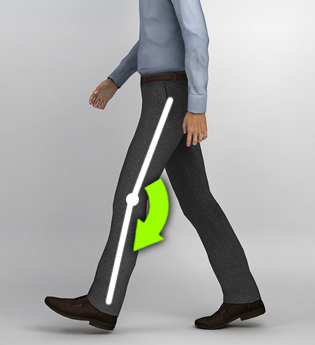 | 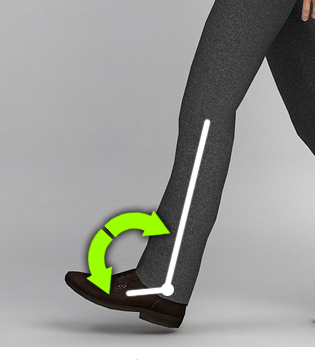 |
| **(Y_6_) Pelvic Tilt [ROM:8°]** | **(Y_9_) Hip Flexion/ Extension**  **[ROM:61°]** | **(Y_12_) Knee Flexion/ Extension**  **[ROM:74°]** | **(Y_15_) Ankle Dorsi/ Plantarflexion**  **[ROM:34°]** |
| **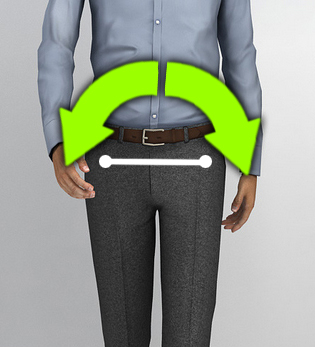** | **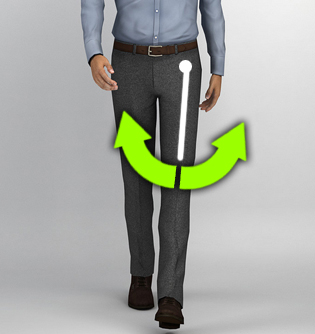** | **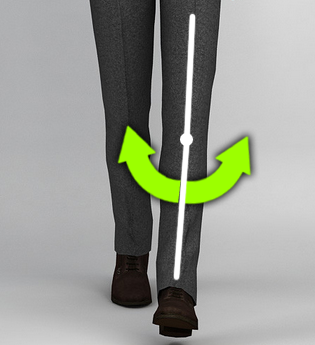** | **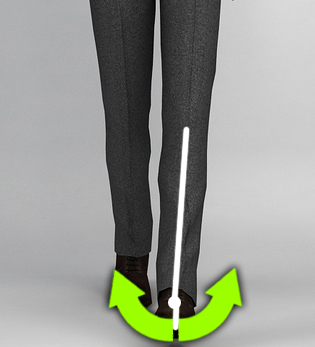** |
| **(Y_7_) Pelvic Obliquity**  **[ROM:12°]** | **(Y_10_) Hip Abduction / Adduction**  **[ROM:16°]** | **(Y_13_) Knee Abduction / Adduction**  **[ROM:22°]** | **(Y_16_) Ankle Inversion/ Eversion**  **[ROM:20°]** |
| **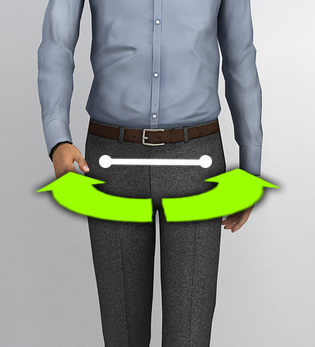** | **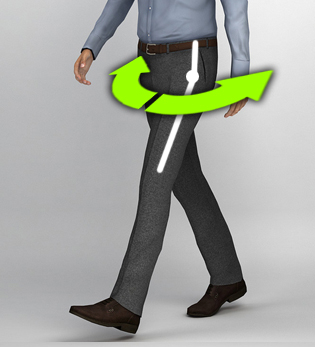** | **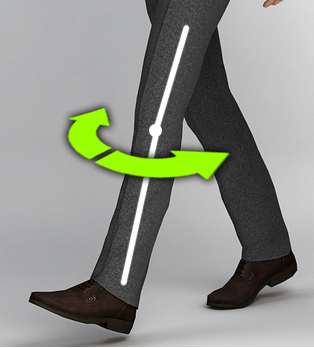** | **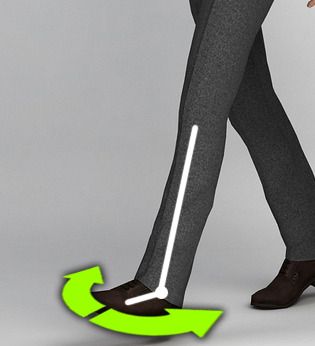** |
| **(Y_8_) Pelvic Rotation**  **[ROM:10°]** | **(Y_11_) Hip Rotation**  **[ROM:25°]** | **(Y_14_) Knee Rotation**  **[ROM:26°]** | **(Y_17_) Ankle Rotation**  **[ROM:22°]** |
|  |  |  | 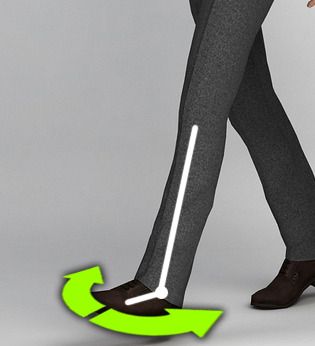 |
|  |  |  | **(Y18) Foot Tilt Vs. Horizontal**  **[ROM:80°]** |

**Figure A.2:** Lower extremity joint angles (Gait parameters Y_6_ to Y_18_)

Note: Range of motion (ROM) values indicated are for subject 12.
